# Analysis of 10 km swimming performance of elite male and female open-water swimmers

**DOI:** 10.1186/2193-1801-2-603

**Published:** 2013-11-12

**Authors:** Pascale Vogt, Christoph Alexander Rüst, Thomas Rosemann, Romuald Lepers, Beat Knechtle

**Affiliations:** Institute of General Practice and for Health Services Research, University of Zurich, Zurich, Switzerland; INSERM U1093, Faculty of Sport Sciences, University of Burgundy, Dijon, France; Gesundheitszentrum St. Gallen, St. Gallen, Switzerland; Facharzt FMH für Allgemeinmedizin, Gesundheitszentrum St. Gallen, Vadianstrasse 26, 9001 St. Gallen, Switzerland

**Keywords:** Elite swimmer, Open-water, Ultra-distance, Performances, Sex difference

## Abstract

This study investigated trends in performance and sex difference in swimming speed of elite open-water swimmers at FINA 10 km competitions (*i.e.* World Cup races, European Championships, World Championships and Olympic Games). Swimming speed and sex difference in swimming speed of the fastest and the top ten women and men per event competing at 10 km open-water races between 2008 and 2012 were analysed using single and multi-level regression analyses. A total of 2,591 swimmers (*i.e.* 1,120 women and 1,471 men) finished 47 races. Swimming speed of the fastest women (1.35 ± 0.9 m/s) and men (1.45 ± 0.10 m/s) showed no changes across years. The mean sex difference in swimming speed for the fastest swimmers was 6.8 ± 2.5%. Swimming speed of the top ten female swimmers per event was 1.34 ± 0.09 m/s and remained stable across the years. The top ten male swimmers per event showed a significant decrease in swimming speed over time, even though swimming speed in the first race (*i.e.* January 2008, 1.40 ± 0.0 m/s) was slower than the swimming speed in the last race (*i.e.* October 2012, 1.50 ± 0.0 m/s) (*P* < 0.05). To summarize, swimming performances remained stable for the fastest elite open-water swimmers at 10 km FINA competitions between 2008 and 2012, while performances of the top ten men tended to decrease. The sex difference in swimming speed in elite ultra-swimmers (~7%) appeared smaller compared to other ultra-distance disciplines such as running. Further studies should examine how body shape and physiology of elite open-water ultra-distance swimmers influence performances.

## Background

Over the last decades, the popularity of ultra-endurance events has considerably increased (Eichenberger et al. 2012a, 2012b; Knechtle et al. 2011; VanHeest et al. 2004). Several studies investigated the participation and performance trends in ultra-endurance events such as ultra-running (Hoffman et al. 2010; Knechtle et al. 2012), ultra-triathlon (Knechtle et al. 2011; Lepers et al. 2013; Rüst et al. 2012) and ultra-swimming (Eichenberger et al. 2012a, 2012b, 2013).

Popularity and participation were growing in specific ultra-endurance sports such as ultra-swimming (Eichenberger et al. 2012a, 2013; VanHeest et al. 2004). The number of ultra-swimmers participating in the ‘English Channel Swim’ has considerably increased since 1900 (Eichenberger et al. 2012a). Swimming as an open-water ultra-distance event became even more popular since its inclusion as a discipline at the 2008 Olympic Games in Beijing (FINA Fédération Internationale de Natation 2013a, http://www.fina.org). Open-water swimming involves many specific challenges that are unique, such as swimming for long duration in cold water (VanHeest et al. 2004).

Performance in open-water ultra-swimmers is influenced by different parameters. Previous studies examined the relationship between anthropometric characteristics and open-waterultra-swimming performance (Knechtle et al. 2010a, 2010b; VanHeest et al. 2004). Anthropometric characteristics showed no relationship to race time with the exception for body mass index in men (Knechtle et al. 2010a, 2010b). Moreover, swimming speed in training was associated with race time (Knechtle et al. 2010a, 2010b). VanHeest et al. (2004) investigated physical and metabolic characteristics of elite open-water ultra-distance swimmers and compared the results with characteristics of elite pool swimmers. Open-water ultra-distance swimmers were smaller and lighter compared to pool swimmers (VanHeest et al. 2004).

Nevertheless, little is known about open-water ultra-distance swimming performance and sex difference in performances (Eichenberger et al. 2012a, 2012b, 2013; Fischer et al. 2013; Tanaka & Seals 1997). Especially, there are no data about performance trends of elite athletes in open-water ultra-distance swimming. So far, only performance trends of recreational athletes have been investigated. For example, Eichenberger et al. (2012a) reported for recreational open-water ultra-distance swimmers at the ‘English Channel Swim’ (32 km) that women were able to achieve a similar performance to men and might have an advantage in long-distance swimming. In contrast, a study from Eichenberger et al. (2013) investigated the performance trends and sex difference of recreational open-water swimmers at the 26.4 km ‘Marathon Swim’ held in Lake Zurich, Switzerland. Performance and sex difference in performance remained stable during the last decades at ~11.5% and it seemed unlikely that female open-water ultra-distance swimmers would achieve the same performance like male swimmers (Eichenberger et al. 2013).

In contrast to recreational ultra-distance swimmers, elite swimmers generally strive to achieve their best performance at the Olympic Games, which is the premier event on the international swimming calendar and the most televised and media covered sport event in the world (Issurin et al. 2008; Trewin et al. 2004). A long held view is that elite swimmers improve their performance between competitions to swim fastest at the Olympic Games and to obtain the best chance of winning a race (Trewin et al. 2004). Given this, elite open-water swimmers are trained professionally by personal coaches and undertake an intense prerace preparation before competing at the Olympic Games (Issurin et al. 2008). Elite’s professional training and therefore high level of performance might lead to a small sex difference in performance. Moreover, female elite swimmers who compete on a high level might be able to perform close to their male counterparts by taking advantages of drafting strategies. Knowing the sex difference in elite open-water swimmers may inspire coaches to find new tactical approaches to prepare their athletes for international events. Pyne et al. (2004) studied the progression in performance of Olympic pool swimmers in freestyle (*i.e.* 50 m, 100 m, 200 m, 400 m, 800 m), backstroke, breaststroke, butterfly (*i.e.* 100 m, 200 m) and medley (*i.e.* 200 m, 400 m) events during a 12-months period resulting to the 2000 Olympic Games. They showed that mean performance improved progressively over the examined time. However, this progression in performance has not yet been investigated and documented in open-water ultra-distance swimmers competing at the Olympic Games.

In this context, the aim of the present study was to investigate the swimming performance and corresponding sex difference of elite open-water swimmers competing in all FINA (Fédération Internationale de Natation) 10km swimming competition held between 2008 and 2012 such as European Championships, World Championships, World Cup races and Olympic Games. Based on the existing literature, we hypothesised that (1) elite female and male open-water ultra-distance swimmers would improve their performances between the 2008 Olympic Games in Beijing and the 2012 Olympic Games in London, (2) the fastest swimming speeds would be achieved at the Olympic Games and (3) the sex difference in 10 km swimming performance would be lower compared to other ultra-distance disciplines such as running.

## Materials and methods

All procedures used in the study met the ethical standards of the Swiss Academy of Medical Sciences and were approved by the Institutional Review Board of Kanton St. Gallen, Switzerland, with a waiver of the requirement for informed consent of the participants given the fact that the study involved the analysis of publicly available data.

### The competitions

Open-water swimming takes place in outdoor bodies, such as rivers, lakes or oceans (http://www.fina.org). There are several competitions held in open-water swimming. The European Championships in open-water swimming are organised by the LEN (Ligue Européenne de Natation 2013), which oversees the aquatic sports in Europe and is affiliated to the FINA (Fédération Internationale de Natation) (http://www.len.eu). The FINA organises the international competitions and World Championships in open-water swimming and defines the rules and regulations for all the events (FINA 2013c, http://www.fina.org). There are annual Championships like the 10km Marathon Swimming World Cup (FINA Fédération Internationale de Natation 2013d, http://www.fina.org). The 10 km World Cup is a series of open-water swimming competitions annually held since 2007 (FINA Fédération Internationale de Natation 2013d, http://www.fina.org). The biennial World Championship in open-water swimming is the largest FINA event and covers race distances of 5 km, 10 km and 25 km (http://www.fina.org). The 10 km World Championship race also serves as a qualifying event for the Olympic 10 km Marathon at the Olympic Games (FINA Fédération Internationale de Natation 2013d, http://www.fina.org). Only 25 female and 25 male athletes who are eligible to participate at the official FINA competitions are able to qualify for the Olympics (FINA Fédération Internationale de Natation 2013d, http://www.fina.org). The first Olympic 10km Marathon was held 2008 in Beijing (FINA Fédération Internationale de Natation 2013b, http://www.fina.org). The qualification of the 25 swimmers followed by virtue of their placing at the 2008 FINA World Open Water Swimming Championship in Seville, Spain and the ‘Good Luck Beijing’ Olympic 10 km Marathon Swim Qualification Race in Beijing, China (FINA Fédération Internationale de Natation 2013b, http://www.fina.org).

The qualifications for the 2012 Olympic Games in London were conducted at the 2011 FINA World Open Water Swimming Championship in Shanghai, China, and at the 2012 FINA Olympic Marathon Swim Qualification Race in Setubal Bay, Portugal (FINA Fédération Internationale de Natation 2013b, http://www.fina.org). In both qualification processes, the ten best athletes from the 10 km World Championships and the nine best ranked athletes from the qualifier races obtained a place. In addition, one athlete from the host country (*i.e.* China in 2008 and Great Britain in 2012) and five athletes as continental representatives could participate (FINA Fédération Internationale de Natation 2013d, http://www.fina.org). The Olympic 10 km Marathon Swim in Beijing was held in a rowing basin on the 20^th^ and 21^st^ of August 2008. The second Olympic race took place in the Serpentine, a recreational lake in Hyde Park in London, on the 9^th^ and 10^th^ of August 2012 (Openwaterpedia 2013, http://www.openwaterpedia.com).

### Data sampling and data analysis

The data set for this study was obtained from the race website http://www.fina.org. This database records every result of any FINA swimming competition held worldwide. Results were available from 10 km swimming events like FINA World Cups, World Championships, European Championships and Olympic Games. In total data were available from 2,719 swimmers (*i.e.* 1,171 women and 1,548 men) who finished in a total of 55 open-water swimming events between 2008 and 2012. After exclusion a total of 2,591 swimmers (*i.e.* 1,120 women and 1,471 men) were analysed in 47 events regarding swimming speed and sex difference in swimming speed. The events were sorted by date and assigned to a ‘relative event date’, expressed in months since January 2008 (*e.g.* January 2008 = 1, February 2008 = 2, March 2008 = 3, etc.). For clarity, events held in the same month were arranged with a separate event number in the figures but treated as parallel events with the same “relative event date” for regression analyses.

To increase the comparability with similar analysis, all race times were converted to swimming speed prior to analysis. Swimming speed was calculated using the equation [race distance in m] / [race time in s] = [swimming speed in m/s]. To get the results as exact as possible, converting and further calculations were performed correct to ten decimal places.

From every event the top (*e.g.* fastest swimming speed) and the top ten (*e.g.* ten fastest swimming speeds) women and men were determined and analysed regarding a potential change of performance over time. Additionally, all events were categorised as ‘World Cup races’, ‘European Championship’, ‘World Championship’ and ‘Olympic Games’. From every category the top ten (*e.g.* ten fastest swimming speeds) women and men ever were determined and compared regarding their swimming speed. The distribution of the different nations was analysed for all finishers, top ten and the winners from every event. To facilitate reading and increase comparability the number of finishers was transformed to a relative value showing the percent of all finishers within the respective category (*e.g.* top or top ten per event). The sex difference in swimming speed was calculated using the equation ([swimming speed in women] – [swimming speed in men]) / [swimming speed in men] × 100. The sex difference in swimming speed was calculated for every pairing of equally placed athletes (*e.g.* between woman and man 1^st^ place, between woman and man 2^nd^ place, etc.) before calculating mean value and standard deviation of all the pairings. In order to facilitate reading, all sex differences in swimming speed were transformed to absolute values before analysing. The density in swimming performance between the 1^st^ and the 10^th^ place as well as between the 1^st^ and the last place was calculated for women and men using the equation ([swimming speed of the 10^th^ place/last place] – [swimming speed of the 1^st^ place])/[swimming speed of the 1^st^ place] × 100. The performance density shows the difference in swimming speed between the winner and the 10^th^ place as well as between the winner and the last finisher expressed as per cent of the winner’s speed in order to indicate the density of the ten fastest athletes as well as the density of all finishers.

### Statistical analysis

Data in the text and figures are given as mean ± standard deviation (SD). In order to increase the reliability of data analyses, each set of data was tested for normal distribution as well as for homogeneity of variances prior to statistical analyses. Normal distribution was tested using a D’Agostino and Pearson omnibus normality test and homogeneity of variances was tested using a Levene’s test. Single and multi-level regression analyses were used to investigate changes in performance of the finishers. A hierarchical regression model was used to avoid the impact of a cluster-effect on results in case one athlete finished more than once in the top one or top ten per event for the analysis of the top and top ten athletes per event regarding the analysis of overall performance as well as of sex difference. As independent variable the ‘relative event date’ was used. To find differences between two groups, a Student’s *t*-test was used in case of normal distributed data (with Welch’s correction in case of unequal variances) and a Mann–Whitney test was used in case of not normal distributed data. Multiple groups were tested for significant differences using a one-way analysis of variance (ANOVA) with subsequent Tukey-Kramer multiple comparison test (family-wise significance and confidence levels 0.05). Statistical analyses were performed using IBM SPSS Statistics (Version 21, IBM SPSS, Chicago, IL, USA) and GraphPad Prism (Version 6.01, GraphPad Software, La Jolla, CA, USA). Significance was accepted at *P* < 0.05 (two-sided for *t*-tests).

## Results

### Finishers

**Figure 1:**
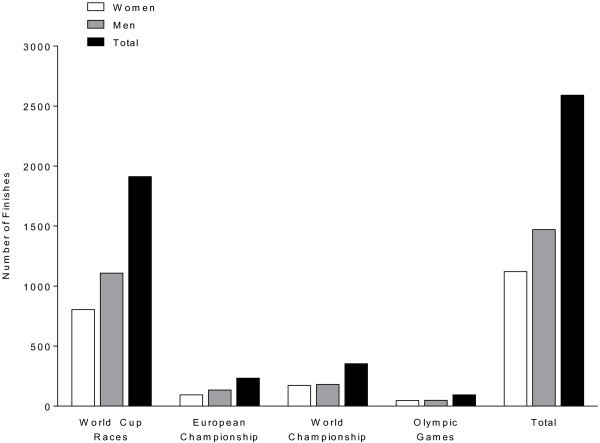
**Number of finishes for the different types of competitions and the total number of finishes of all events.**

**Figure 2:**
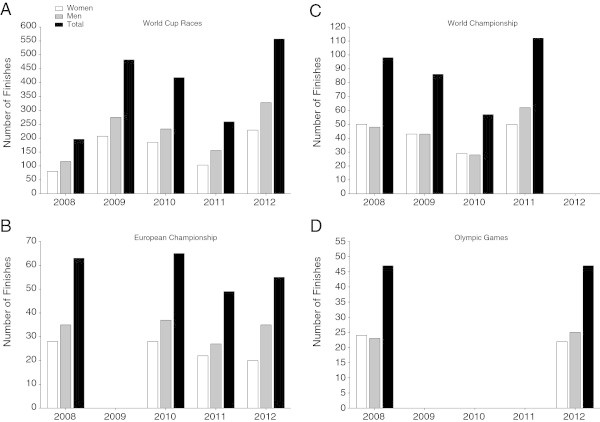
**Number of finishes in the different types of competitions from 2008 to 2012 for World Cup races (Panel A), World Championship (Panel B), European Championship (Panel C), and Olympic Games (Panel D).**

In total, data were available from 1,171 women and 1,548 men finishing at the FINA World Cup races, European Championships, World Championships and Olympic Games. From the FINA Swimming World Cup a total of eight races had to be excluded from analyses due to too small numbers of finishers for statistical analyses, leading to an exclusion of 51 women and 77 men in the FINA World Cup races. The athletes started in a total of 55 events, whereof eight were excluded, leading to a number of 47 analysed events between January 2008 and October 2012. Between the first race in January 2008 (*i.e.* FINA Swimming World Cup in Santos) and the last race in October 2012 (*i.e.* FINA in Shantou), a total number of 2,591 swimmers (1,120 women and 1,471 men) finished in 47 analysed events (Figure 1). Figure 2 illustrates the number of finishes in the different competitions-types during the studied period. A total of 804 women and 1,108 men finished at the FINA World Cup races, 172 women and 181 men at the World Championships, 98 women and 134 men at the European Championships and 46 women and 48 men at the Olympic Games (Figure 2).

**Figure 3:**
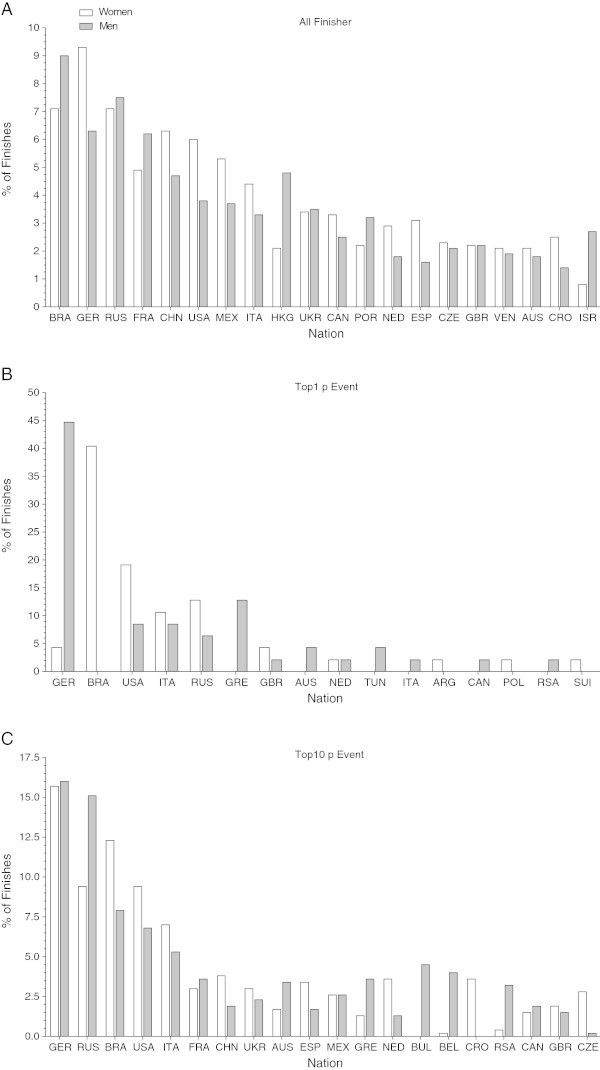
**Percentage of finishes among the different nationalities for all finishers (Planel A), top (Planel B) and top ten (Planel C) women and men in all events.** In Panel **A** and **C** only the 20 nationalities with the highest number of finishers are illustrated. ARG: Argentina, AUS: Australia, BEL: Belgium, BRA: Brazil, BUL: Bulgaria, CAN: Canada, CHN: China, CRO: Croatia, CZE: Czechoslovakia, ESP: Spain, FRA: France, GBR: Great Britain, GER: Germany, GRE: Greece, HKG: Hong Kong, ISR: Israel, ITA: Italy, MEX: Mexico, NED: Netherlands, POL: Poland, POR: Portugal, RSA: Republic of South Africa, RUS: Russia, SUI: Switzerland, TUN: Tunisia, UKR: Ukraine, USA: United States of America, VEN: Venezuela.

Figure 3 shows the distribution of the 20 nationalities with the highest number of finishers. From a total of 2,591 finishers (1,120 women, 1,471 men), most of the finishers originated from Brazil (213) followed by athletes from Germany (197) and Russia (190). The percentage of female finishes was higher for German swimmers (9.3%) compared to Russian (7.1%) and Brazilian swimmers (7.1%) (Figure 3A). For men, the percentage of finishes was higher in Brazilian (9.0%) and Russian (7.5%) but lower in German athletes (6.3%) (Figure 3A). Considering the fastest female and male swimmers in the 47 analysed events, most of the swimmers were from Germany (23), Brazil (19) and the USA (13). In women, the percentage of finishes was higher in Brazilian (40.4%) and American (19.1%) but lower in German swimmers (4.3%). In men, most of the finishers were from Germany (44.7%), whereas no finishes from Brazil (0.0%) were recorded (Figure 3B). When comparing the 20 nationalities with the highest number for the ten fastest swimmers per event, most of the finishers originated from Germany (149), followed by Russia (115) and Brazil (95) (Figure 3C). In both sexes the highest percentage of finishers were from Germany (women 15.7%, men 16.0%). Whereas in women the percentage of finishes was higher in Brazilian (12.3%) compared with Russian swimmers (9.4%), in men the percentage of finishes was higher in Russian (15.1%) compared with Brazilian swimmers (7.9%) (Figure 3C).

Respecting multiple finishes in the fastest women and men per event, a total of 14 women and 17 men finished totally 47 times each. Most of the finishers finished once or twice, whereas one woman and one man finished even more than ten times during the time period (Table 1). Considering the ten fastest athletes per event, 117 women and 119 men competed 470 times each. Most of the finishers finished between one and three times ever, but more than ten women and men finished more than ten times during the analysed time period (Table 2).

**Table 1:** **Number of the fastest female and male finishers and the number of finishes between 2008 and 2012 for the 47 events**

	Women	Men
Finishers	14	17
Finishes	47	47
1 Finish	5	11
2 Finishes	4	3
3 Finishes		
4 Finishes	1	1
5 Finishes	1	
6 Finishes	1	1
7 Finishes		
8 Finishes	1	
9 Finishes		
10 Finishes		
>10 Finishes	1	1

Additionally, information about the number of finishers with more than one finish is given.

**Table 2:** **Number of top ten female and male finishers and the number of finishes in the 47 events between 2008 and 2012**

	Women	Men
Finishers	117	119
Finishes	470	470
1 Finish	45	51
2 Finishes	22	23
3 Finishes	17	15
4 Finishes	8	4
5 Finishes	2	4
6 Finishes	4	4
7 Finishes	3	4
8 Finishes	4	2
9 Finishes		
10 Finishes	2	
>10 Finishes	10	12

Additionally, information about the number of finishers with more than one finish is given.

### Performance trends and sex difference in swimming speed

**Figure 4:**
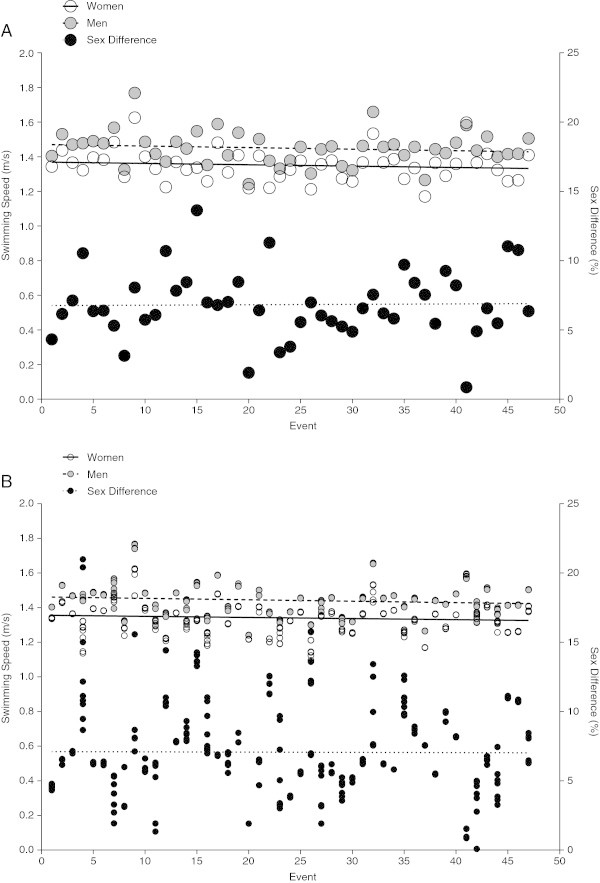
**Change in swimming speed of top (Panel A) and top ten (Panel B) women and men with sex difference across events.**

For both women and men, the fastest swimming speed did not change significantly over time (Table 3). The mean swimming speed of the fastest women across the 47 events between 2008 and 2012 was 1.35 ± 0.09 m/s (Figure 4A). Men completed with a mean swimming speed of 1.45 ± 0.10 m/s. The mean sex difference in swimming speed was 6.8 ± 2.5% and showed no change over time, also when controlled for multiple finishes (Figure 4A, Table 3). The ten fastest men per event achieved a mean swimming speed of 1.44 ± 0.10 m/s during the analysed period (Figure 4B). Mean swimming speed for the ten fastest women remained stable across the 47 events at 1.34 ± 0.09 m/s (Figure 4B, Table 3). The equivalent male statistic displayed a significant downward trend (*P* < 0.05), even though the recorded swimming speed in the first race in January 2008 was with 1.40 ± 0.0 m/s slower than the swimming speed in the last race in October 2012 (1.50 ± 0.0 m/s). Correction for multiple inclusion of the same athlete showed no alteration in this result (Table 3). The sex difference in swimming speed remained unchanged at 7.06 ± 2.8% (Figure 4B), also when corrected for multiple finishes of the same athlete (Table 3).

**Table 3:** **Multi-level regression analyses for the change in performance across years for women and men (Model 1) with correction for multiple finishes (Model 2)**

Model	***β***	SE ( ***β*** )	Stand. ***β***	T	***p***
**Change in swimming speed for the fastest women per event**					
1	-0.001	0.001	-0.100	-0.674	0.504
2	-0.001	0.001	-0.100	-0.674	0.504
**Change in swimming speed for the fastest men per event**					
1	-0.001	0.001	-0.106	-0.713	0.479
2	-0.001	0.001	-0.106	-0.713	0.479
**Change in sex difference in swimming performance for the fastest swimmers per event**					
1	0.005	0.022	0.034	0.225	0.823
2	0.005	0.022	0.034	0.225	0.823
**Change in swimming speed for the ten fastest women per event**					
1	0.000	0.000	-0.079	-1.706	0.089
2	0.000	0.000	-0.079	-1.706	0.089
**Change in swimming speed for the ten fastest men per event**					
1	-0.001	0.000	-0.096	-2.079	0.038
2	-0.001	0.000	-0.096	-2.079	0.038
**Change in sex difference for the ten fastest swimmers per event**					
1	0.001	0.008	0.008	0.179	0.858
2	0.001	0.008	0.008	0.179	0.858

### Performance density of finishers

**Figure 5:**
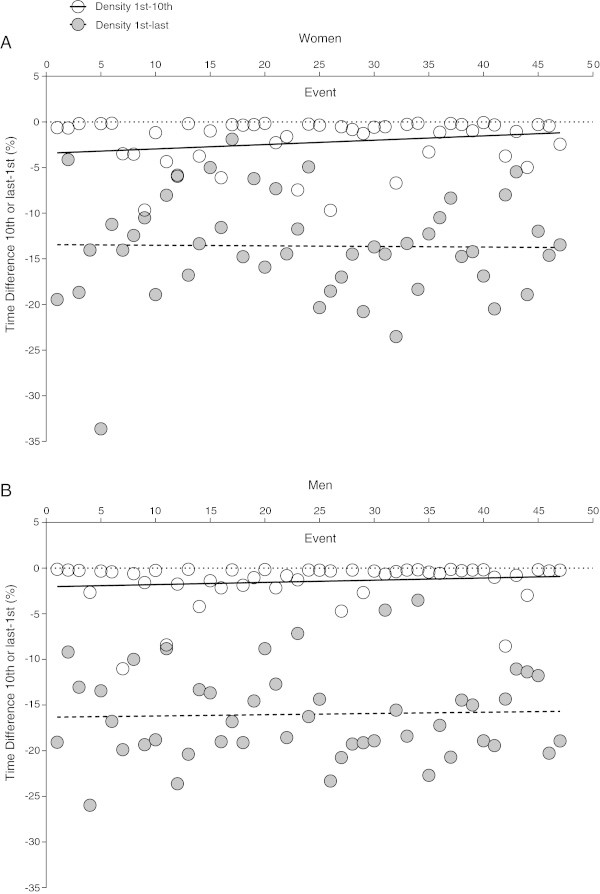
**Difference in swimming speed between the winner and the 10th placed athlete (Panel A) and the winner and the last placed athlete expressed as percentage of the winner time (Panel B) for women and men across all 47 events.**

The swimming speed difference per event between the winner and the 10^th^ place as well as the winner and the last place is presented in Figure 5. The mean swimming speed difference between the first and 10^th^ finisher in women was 2.3 ± 3.1% per event. The swimming speed difference between the first and the last finisher in women was 13.6 ± 5.9%. Between the first and 10^th^ finisher as well as between the first and last finisher, no significant change in swimming speed difference could be observed across all events, also when controlled for multiple finishes of the same athlete (Figure 5A and Table 4). Mean swimming speed difference between the first and 10^th^ finisher in men was 1.5 ± 2.4% per event. Swimming speed difference between the first and last finisher in men was 16.0 ± 5.0%. Swimming speed difference between the first and 10^th^ finisher as well as between the first and last finisher remained unchanged across all events, also when controlled for multiple finishes of the same athlete (Figure 5B and Table 4).

**Table 4:** **Regression analyses for the change in density across years for women and men**

	***β***	SE ( ***β*** )	Stand. ***β***	T	***p***
**Density 1** ^**st**^ **to 10** ^**th**^					
Women	0.034	0.027	0.185	1.262	0.214
Men	0.018	0.021	0.131	0.889	0.379
**Density first to last**					
Women	-0.003	0.052	-0.009	-0.061	0.951
Men	0.013	0.044	0.045	0.300	0.766

### Comparison of performance at different events

**Figure 6:**
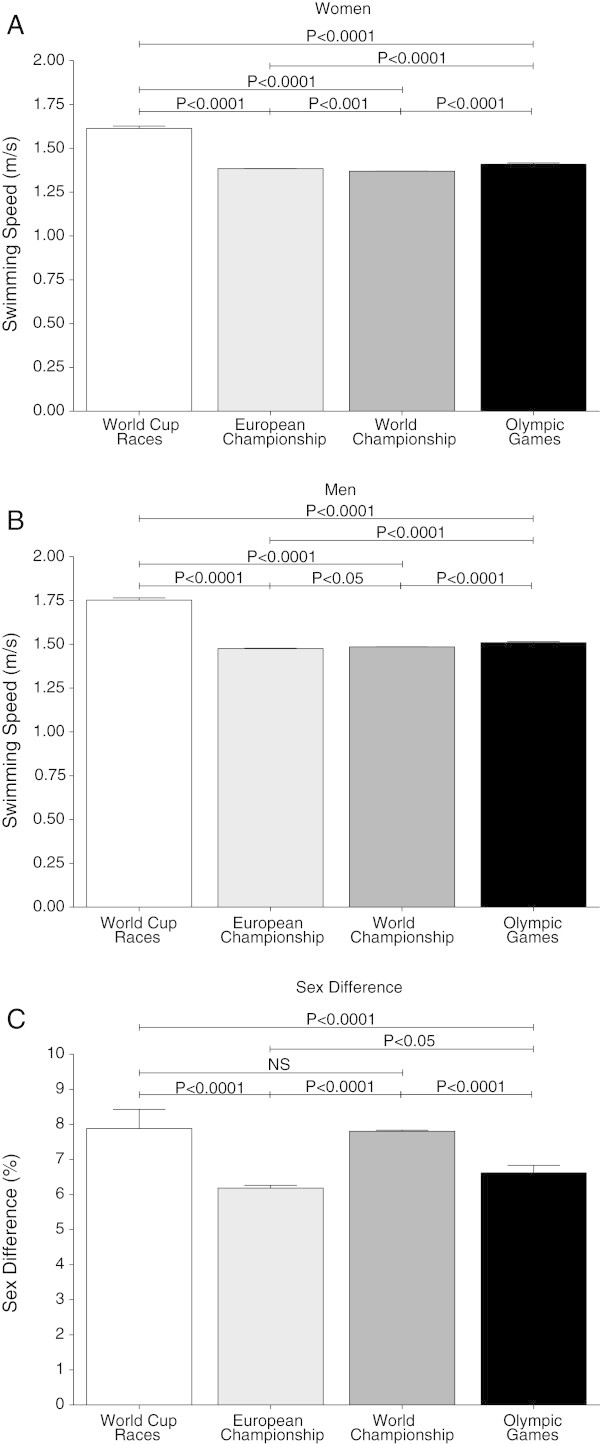
**Comparison of the ten fastest women (Panel A) and men (Panel B) and the sex difference (Panel C) in World Cup races, European Championship, World Championship and Olympic Games.**

The ten fastest women per type of competition showed a significant higher swimming speed at the FINA World Cup races (1.61 ± 0.01 m/s) than at the European Championships (1.38 ± 0.0 m/s) (*P* < 0.01), at the World Championships (1.37 ± 0.0 m/s) (*P* < 0.01) or at the Olympic Games (1.41 ± 0.01 m/s) (*P* < 0.01). Swimming speed at the Olympic Games was significantly faster than at the European Championships (*P* < 0.01) or at the World Championships (*P* < 0.01), whereas swimmers at the World Championships were even slower than swimmers at the European Championships (*P* < 0.01) (Figure 6A). In men, the ten fastest finishes at the World Cup races were with 1.75 ± 0.01 m/s faster than the ten fastest finishes at the European Championships with 1.48 ± 0.0 m/s *P* < 0.01), at the World Championships with 1.49 ± 0.01 m/s (*P* < 0.01) and at the Olympic Games with 1.51 ± 0.01 m/s (*P* < 0.01). Swimmers at the Olympic Games were significantly faster than swimmers at the European Championships (*P* < 0.01) or World Championships (*P* < 0.01). Swimming speed in World Championship was significantly faster than in European Championships (*P* < 0.05) (Figure 6B).

The sex difference in swimming speed showed no significant difference between World Cup races (7.9 ± 0.6%) and World Championships (7.8 ± 0.0%), whereas the sex difference in swimming speed in World Cup races was significant higher than in European Championships (6.2 ± 0.1%) (*P* < 0.01) or at the Olympic Games (6.6 ± 0.2%) (*P* < 0.01). There was also a significant higher sex difference in swimming speed in World Championships compared to European Championships (*P* < 0.01) or to Olympic Games (*P* < 0.01), whereas sex difference at the Olympic Games was higher than at the European Championships (Figure 6C).

### Swimming performances at the 2008 vs. the 2012 Olympic Games

**Figure 7:**
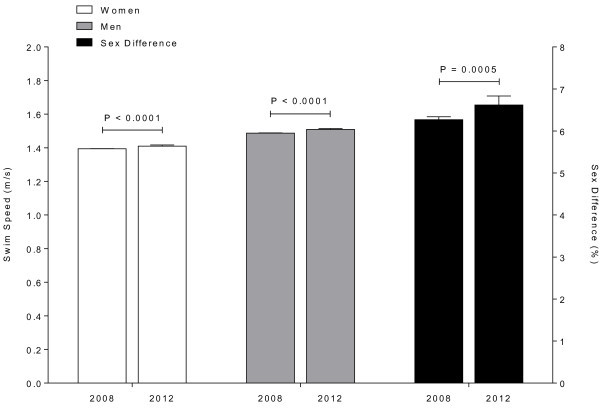
**Comparison of the ten fastest swimming speeds in women and men and the sex difference between the Olympic Games 2008 and 2012.**

Figure 7 shows the comparison of swimming speeds at the 2008 Olympic Games in Beijing and 2012 in London. The mean of the ten fastest swimming speeds in women and men and the sex difference in swimming speed differed significantly between the two competitions (*P* < 0.01). Female and male swimmers achieved a faster swimming speed at the 2012 Olympic Games. The ten fastest women showed a swimming speed of 1.39 ± 0.00 m/s at the 2008 Olympic Games and enhanced their swimming speed to 1.41 ± 0.01 m/s at the 2012 Olympic Games. Men also improved their swimming speed from 1.49 ± 0.00 m/s in 2008 to 1.51 ± 0.01 m/s in 2012. The sex difference in swimming speed increased from 6.3 ± 0.1% to 6.6 ± 0.2% (*P* < 0.01) (Figure 7).

## Discussion

The aim of the present study was to examine the performance trends and sex difference in swimming speed in elite open-water athletes at the FINA 10 km competitions including the Olympic Games from 2008 to 2012. The results showed that swimming speed for the fastest female and male swimmers remained stable during the studied period. The sex difference in swimming speed was ~7%, which is lower compared with the sex difference in other ultra-distance disciplines such as running. The top ten men per event showed a significant decrease in swimming speed during the studied period. The comparison of performances in the different competitions showed that the top ten women and men achieved their fastest speeds at the World Cup races. Regarding the performances of Olympic athletes, swimming speed increased significantly for both sexes between the 2008 Olympic Games in Beijing and the 2012 Olympic Games in London.

### Performance trends and sex difference in swimming speed

Swimming speed for the fastest female and male swimmer remained stable between 2008 and 2012. The changes in swimming speed for the fastest athletes also showed no alteration when controlled for multiple finishes. An important finding was the small sex difference in swimming speed of ~7%. The fact that elite athletes were investigated partially might explain this finding. Elite open-water swimmers are trained professionally by personal trainers and undertake an intense pre-competitive preparation (Issurin et al. 2008). Therefore elite athletes perform on a high level and show a low variability in performance (Issurin et al. 2008).

Generally, the best male athlete seemed always to perform better (*i.e.* swim faster) than the best female athlete (Fischer et al. 2013; Knechtle et al. 2011; Lepers et al. 2013; Lepers & Maffiuletti 2011). There are several explanations for the faster swimming speed of male athletes in ultra-endurance swimming. Male swimmers have a greater aerobic capacity (VO_2_max) compared to female swimmers (Eichenberger et al. 2013; Lepers 2008). Furthermore, men have more androgen (Lynch & Hoch 2010; Eichenberger et al. 2013) and more skeletal muscle mass (Lynch & Hoch 2010; Lepers 2008).

Nevertheless, the sex difference in swimming speed of ~7% in these elite open-water ultra-distance swimmers seemed lower compared to the sex difference of athletes in other ultra-endurance disciplines such as ultra-running (Lepers et al. 2013; Rüst et al. 2013a) or ultra-cycling (Rüst et al. 2013b). For example, Coast et al. (2004) investigated the world best running performances at distances from 100 m to 200 km and reported an average sex difference in running speed of ~12%. Rüst et al. (2013a) examined running performances in 100mile ultra-marathons held between 1998 and 2011 and found an average sex difference in running speed of ~17%. In ultra-cycling, such as the ‘Race Across America’, a sex difference in cycling speed of ~19% between the annual fastest women and men was described (Rüst et al. 2013b).

Accordingly, Knechtle et al. (2011) found that the sex difference in Double and Triple Iron ultra-triathlon was the smallest in swimming compared to cycling and running. The sex difference between the different locomotion modes was also analysed in previous studies at the ‘Ironman Hawaii’ triathlon covering 3.8 km swimming, 180 km cycling, and 42 km running (Lepers 2008; Lepers & Maffiuletti 2011; Lepers et al. 2013). The sex difference in swimming (~10-12%) tended to be smaller compared to cycling (~13-15%) and running (~13-18%) (Lepers 2008; Lepers & Maffiuletti 2011; Lepers et al. 2013).

A possible reason for the lower sex difference in swimming compared to cycling and running could be the difference in body fat percentage between women and men, with 22-26% body fat for female and 13-16% for male athletes (Eichenberger et al. 2013; Lavoie & Montpetit 1986; Lepers 2008). While high body fat is a disadvantage in weight-bearing activities such as running (Lepers 2008); it seemed to improve buoyancy in water (Lepers 2008). Furthermore, higher body fat in female swimmers provided a better isolation against cold water (Etter et al. 2013; Knechtle et al. 2009). In addition, it has been shown that underwater torque was lower for women than for men and mechanical efficiency for women was greater compared to men (Lepers 2008; Lepers & Maffiuletti 2011). Factors leading to a higher mechanical efficiency for women are their smaller body size (*i.e.* a smaller body drag) (Lepers & Maffiuletti 2011; Tanaka & Seals 1997), their smaller body density (*i.e.* more body fat percentage) (Lepers & Maffiuletti 2011; Tanaka & Seals 1997) and their shorter legs (Lepers & Maffiuletti 2011; zzTanaka & Seals 1997), resulting in a more horizontal and streamlined position (Etter et al. 2013; Lepers & Maffiuletti 2011; Tanaka & Seals 1997).

The small sex difference in swimming speed might also be explained by the fact that the athletes competed in ultra-distance swimming events. It was described that the sex difference in swimming speed decreased with increasing swimming distances, whereas in cycling (Lepers 2008) and running (Tanaka & Seals 1997) the gap between women and men appeared constant with increasing race distance (Hoffman 2008; Lepers 2008; Tanaka & Seals 1997). Indeed, previous studies showed that the sex difference increased with longer running distances (Coast et al. 2004; Knechtle et al. 2011). Contrary to running events, Tanaka and Seals (1997) showed a progressively smaller sex difference with increasing swimming distance from 50 m (19%) to 1,500 m (11%).

Regarding the top ten open-water swimmers in our study, swimming speed of the ten fastest women per event and the sex difference in swimming speed (~7%) remained stable across years. Surprisingly, the top ten men showed a decrease in swimming speed across the years, also when controlled for multiple participants. The reasons for this finding are not clear, but training and anthropometric characteristics such as body mass, body height and length of arm have been discussed to influence performance in open-water ultra-distance swimming (Knechtle et al. 2008, 2010a, 2010b). Knechtle et al. (2010a) found that anthropometrical characteristics were not related to open-water ultra-distance performance, except body mass index to male performance. Interestingly, swimming speed in training influenced race time for both sexes (Knechtle et al. 2010a). This was similar to a study with focus on male swimmers (Knechtle et al. 2010b). Anthropometric characteristics were not associated with swimming performance, whereas high speed in training was associated with faster race times (Knechtle et al. 2010b). In contrast, Siders et al. (1993) reported that body height, body weight, body fat and fat free weight influenced performance in female, but not in male sprint swimmers. However, the role of training intensity for performance in open-water ultra-distance swimming, especially in elite athletes, is not yet clarified and should be investigated in additional studies. Finally, physical factors such as overtraining or psychological factors should be considered to influence performances as well (Issurin et al. 2008; Trewin et al. 2004).

### Performance density of finishers

The average swimming speed difference between the first and 10^th^ finisher was relatively small in both sexes, but was slightly greater in men compared to women. The high density could be explained by the professionally trained athletes. Only the world’s best athletes came together and competed in these races. Given this, the ten best athletes were very close to each other and no big differences in performances occurred. The mean swimming speed difference between the first and last finisher was smaller in women compared with men, assuming that the overall female performance density was higher.

Drafting during swimming (*i.e.* swimming directly behind or aside another swimmer) was not controlled, but might have influenced the density of finishers. Hausswirth and Brisswalter (2008) reported that hydrodynamic drag is reduced while swimming in a sheltered position. Given this, drafting allows swimmers to reduce energy cost and gain time for swimming at maximal speed (Hausswirth & Brisswalter 2008). Furthermore, it has been observed that swimming behind a leader been induces changes in speed (*i.e.* swimmers showed a faster velocity) (Hausswirth & Brisswalter 2008). Bentley et al. (2008) examined drafting in Olympic distance triathlon and described that drafting in the swimming and cycling split may result in a better tactical approach to increase overall performance.

### Origin of athletes

Germany and Brazil were the most represented nations when analysing the origin of all finishers. Interestingly, most of the female winners were from Brazil and most of the male winners from Germany. The nationality was analysed for all finishers at World Cup races, European Championships, World Championships and Olympic Games between 2008 and 2012. The different competitions took place all over the world. Interestingly, all 10 km World Cup races were annually held in Santos, Brazil. This fact might have increased popularity of open-water swimming in Brazil and explain the high percentage of Brazilian finishers in this study. The location of the sport event and therefore small travel distance for local athletes seems to play an important role in the decision for participating at a competition (Jürgens et al. 2012). Jürgens et al. (2012) analysed the participation and performance by nationality at the Ironman Switzerland in Zurich. Totally 90% of the participants originated from European countries. Most of the participants and winners originated from Switzerland, followed by Germany. The reasons for Switzerland’s dominating position were that the Ironman Switzerland is a highly prestigious race for Swiss athletes and that the Swiss are familiar with the climate and the terrain Jürgens et al. (2012).

The reason for the high percentage of German finishers, especially in male winners, is not clear. Previous studies have discussed that European athletes dominate participation and performance at endurance events in Europe as well as overseas (Cejka et al. 2013, Knechtle et al. 2013). In a study about 100 km ultra-marathons worldwide the most finishers were from Europe. The greater number of European athletes was explained by the higher popularity of ultra-marathons in Europe compared with other continents (Cejka et al. 2013). In contrast, in international middle and long-running events Kenyan and Ethiopian runners are dominant (Wilber & Pitsiladis 2012). Their success in running was explained by their body composition. Their long and slender legs might be advantageous in biomechanical and metabolic effectiveness (Cejka et al. 2013; Wilber & Pitsiladis , Wilber & Pitsiladis Wilber & Pitsiladis 2012).

### Comparison of performance in the different competitions

When comparing swimming speeds across the different competitions, the top ten women and men achieved their fastest speeds at the World Cup races and not at the Oympic Games. Nevertheless, female and male swimming speed was significantly faster at the Olympic Games compared to both the European and the World Championships. Because the World Cup races consist of a number of competitions and the swimmers participate several times, they might have a higher chance to achieve good results.

In previous studies swimming performances with different strokes (*i.e.* freestyle, butterfly, breaststroke, and backstroke) and distances (*i.e.* 50 m, 100 m, 200 m, 400 m, 800 m, and 1,500 m) before and at the Olympic Games were compared (Issurin et al. 2008; Pyne et al. 2004; Trewin et al. 2004). Trewin et al. (2004) studied the relation and progression between the FINA top-50 world-ranking and Olympic performance in pool swimmers. Overall performance time decreased from the world-ranking to the Olympic Games in both sexes. Surprisingly, most of the Olympic medallists had a top ten world-ranking and were the only swimmers, which could improve their performance (Trewin et al. 2004). Issurin et al. (2008) analysed peaking (*i.e.* obtaining the best performance) in the 2004 Athens Olympic swimming competitions and how it is influenced by factors such as age, sex or swimming stroke-types. Interestingly, also in this study an average performance decline was observed in all the swimming events (Issurin et al. 2008). The athletes failed to improve their prior swimming time in pre-Olympic competitions and competed slower at the 2004 Olympic Games (Issurin et al. 2008). In contrast, Pyne et al. (2004) investigated all competitions from the USA and Australia swimming teams during a 12 months period prior to the 2000 Olympic Games. Swimmers of both teams progressively improved their race time.

An interesting finding was that women had a slower swimming speed in the World Championships than in the European Championships, whereas men showed a slower swimming speed in the European Championships than in the World Championships. Previous studies showed that training variables influence swimming speed in competitive performance (Anderson et al. 2008; Stewart & Hopkins 2000). Stewart and Hopkins (2000) reported a significantly better performance with greater weekly training mileage for swimmers specialised in sprint (*i.e.* 50 m and 100 m) and middle-distance (*i.e.* 200 m and 400 m). Pyne et al. (2004) described that the progression in race times between competitions is related to the effects of training. Training includes improvement of fitness, technique, swimming skills and psychological skills (Pyne et al. 2004).

During a given competition drafting as a pacing strategy (Hausswirth & Brisswalter 2008; Pyne et al. 2004) and stroke-type (Issurin et al. 2008) are important for swimming speed. Additionally, the different circumstances between the events have to be considered. Every open-water swimming competition is unique because of its location and weather condition (VanHeest et al. 2004). For example, swimming in salt water improves buoyancy and this may enhance swimming performance (Stiefel et al. 2013).

### Comparison of swimming performance at the Olympic Summer Games 2008 and 2012

From the 2008 Olympic Games in Beijing to the 2012 Olympic Games in London, swimming speeds improved for both sexes. To date, no study examined the progression and variability of performance of Olympic ultra-distance swimmers between Olympic competitions. Trewin et al. (2004) investigated the relation between world-ranking and Olympic performance in pool swimmers and found that medallists, in contrast to non-medallists, improved their previous best time and swam faster at the Olympic Games. Supposing that the fastest swimmers are capable to improve their performance, might explain why the top ten Olympic swimmers in this study increased their swimming speed between the Olympic Games 2008 in Beijing and 2012 in London. Regarding elite athletes, Charles and Bejan (2009) examined the evolution of world speed record and shape in modern runners and swimmers. The improvement in the 100 m-freestyle swimming world records seemed to be related to the changes in the athlete’s body shape across years. In fact, the fastest sprint swimmers are not only becoming faster but also heavier and taller (Charles & Bejan 2009). VanHeest et al. (2004) examined the anthropometrical characteristics of elite swimmers and found that open-water swimmers were smaller and lighter compared with competitive pool swimmers. Given this, it would be an interesting implication for future studies to investigate the evolution of body shape in Olympic ultra-distance open-water swimmers and whether there is a relation between the fastest swimming speed and body shape.

### Limitations

The study is limited regarding the influence of anthropometric characteristics such as body mass, body height and length of arm on performance (Knechtle et al. 2010a, 2010b). Furthermore, the potential impact of training intensity and training volume on performance was not investigated in this study (Anderson et al. 2008; Knechtle et al. 2010a, 2010b; Stewart & Hopkins 2000). Other factors such as motivation (Houston et al. 2011), changes in fluid metabolism (Wagner et al. 2012), thermoregulation (Kerr et al. 1998; Knechtle et al. 2009; Nuckton et al. 2000), energy metabolism (Weitkunat et al. 2012) and pacing strategies (*e.g.* drafting) during a competition (Hausswirth & Brisswalter 2008; Houston et al. 2011) were not controlled and might have affected performances too.

## Conclusion

During the 2008–2012 period, swimming performances remained stable for the best elite female and male open-water swimmers competing at 10 km FINA events. The top ten male swimmers per event showed a significant decrease in swimming speed during the same period. The sex difference in swimming speed of ~7%, is smaller compared with the sex difference in other disciplines such as ultra-running and ultra-cycling. In the comparison of the different types of competitions, female and male swimmers achieved their fastest swimming speeds at the World Cup races. Interestingly, women showed a slower swimming speed at the World Championships than at the European Championships. Swimming speed between the Olympic Games in 2008 to 2012 increased for female and male athletes. Future investigations should analyse pre-competitive training and the effect of pacing strategies. In addition, further studies are needed to examine how body shape and physiology of elite open-water ultra-distance swimmers influence swimming speed and sex difference in swimming speed.
